# Changing Epidemiology of COVID-19 in Children and Adolescents Over Four Successive Epidemic Waves in South Africa, 2020–2022

**DOI:** 10.1093/jpids/piad002

**Published:** 2023-01-17

**Authors:** Nicola Chiwandire, Waasila Jassat, Michelle Groome, Tendesayi Kufa, Sibongile Walaza, Nicole Wolter, Anne von Gottberg, Heather J Zar, Gary Reubenson, Stefano Tempia, Joy Ebonwu, Nevashan Govender, Genevie Ntshoe, Andronica Moipone Shonhiwa, Lucille Blumberg, Cheryl Cohen

**Affiliations:** National Institute for Communicable Diseases of the National Health Laboratory Service, Johannesburg, South Africa; National Institute for Communicable Diseases of the National Health Laboratory Service, Johannesburg, South Africa; Right to Care, Johannesburg, South Africa; National Institute for Communicable Diseases of the National Health Laboratory Service, Johannesburg, South Africa; School of Pathology, University of the Witwatersrand, Johannesburg, South Africa; National Institute for Communicable Diseases of the National Health Laboratory Service, Johannesburg, South Africa; School of Public Health, University of the Witwatersrand, Johannesburg, South Africa; National Institute for Communicable Diseases of the National Health Laboratory Service, Johannesburg, South Africa; School of Public Health, University of the Witwatersrand, Johannesburg, South Africa; National Institute for Communicable Diseases of the National Health Laboratory Service, Johannesburg, South Africa; School of Pathology, University of the Witwatersrand, Johannesburg, South Africa; National Institute for Communicable Diseases of the National Health Laboratory Service, Johannesburg, South Africa; School of Pathology, University of the Witwatersrand, Johannesburg, South Africa; Department of Paediatrics and Child Health, Red Cross War Memorial Children’s Hospital, Cape Town, South Africa; MRC Unit on Child & Adolescent Health, University of Cape Town, Cape Town, South Africa; Department of Paediatrics and Child Health, Faculty of Health Sciences, University of the Witwatersrand, Rahima Moosa Mother and Child Hospital, Johannesburg, South Africa; National Institute for Communicable Diseases of the National Health Laboratory Service, Johannesburg, South Africa; School of Public Health, University of the Witwatersrand, Johannesburg, South Africa; National Institute for Communicable Diseases of the National Health Laboratory Service, Johannesburg, South Africa; National Institute for Communicable Diseases of the National Health Laboratory Service, Johannesburg, South Africa; National Institute for Communicable Diseases of the National Health Laboratory Service, Johannesburg, South Africa; School of Health Systems and Public Health, University of Pretoria, Pretoria, South Africa; National Institute for Communicable Diseases of the National Health Laboratory Service, Johannesburg, South Africa; National Institute for Communicable Diseases of the National Health Laboratory Service, Johannesburg, South Africa; Right to Care, Johannesburg, South Africa; National Institute for Communicable Diseases of the National Health Laboratory Service, Johannesburg, South Africa; School of Public Health, University of the Witwatersrand, Johannesburg, South Africa

**Keywords:** children, adolescents, COVID-19, South Africa, admissions

## Abstract

**Background:**

South Africa experienced four waves of SARS-CoV-2 infection, dominated by Wuhan-Hu, Beta, Delta, and Omicron (BA.1/BA.2). We describe the trends in SARS-CoV-2 testing, cases, admissions, and deaths among children and adolescents in South Africa over successive waves.

**Methods:**

We analyzed national SARS-CoV-2 testing, case, and admissions data from March 2020 to February 2022 and estimated cumulative rates by age group for each endpoint. The severity in the third versus the fourth wave was assessed using multivariable logistic regression.

**Results:**

Individuals ≤18 years comprised 35% (21,008,060/60,142,978) of the population but only 12% (424,394/3,593,644) of cases and 6% (26,176/451,753) of admissions. Among individuals ≤18 years, infants had the highest admission (505/100,000) rates. Testing, case, and admission rates generally increased successively in the second (Beta) and third (Delta) waves among all age groups. In the fourth (Omicron BA.1/BA.2) wave, the case rate dropped among individuals ≥1 year but increased among those <1 year. Weekly admission rates for children <1 year (169/100,000) exceeded rates in adults (124/100,000) in the fourth wave. The odds of severe COVID-19 in all admitted cases were lower in the fourth wave versus the third wave in each age group, but they were twice as high in admitted cases with at least one comorbidity than those without.

**Conclusions:**

The admission rate for children <5 years was higher in the fourth wave than in previous waves, but the overall outcomes were less severe. However, children with at least one comorbidity had increased odds of severe disease, warranting consideration of prioritizing this group for vaccination.

## INTRODUCTION

South Africa recorded its first case of SARS-CoV-2 in March 2020 and subsequently, the country experienced four successive epidemic waves dominated by Wuhan-Hu, Beta, Delta, and Omicron (BA.1 and BA.2 subvariants). The first two waves saw larger proportions of adults infected compared to children in South Africa and across the world [1, 2]. Even though children were commonly asymptomatic or presented with the mild disease after infection [3], the Delta and Omicron variants have been reported to be associated with an increased proportion of cases in children in many countries including South Africa [4, 5]. Several studies, including from South Africa, have described SARS-CoV-2 epidemiology in children early on in the pandemic, generally describing low attack rates and symptomatic fraction compared to disease in adults [2, 6–9]. Several high-income countries have reported increased hospitalizations among children in the Omicron wave while some reported no change [4, 10–13].

National-level surveillance data from South Africa found that the Omicron wave was associated with reduced clinical severity compared to the previous three waves, but data on children were only included in broad age bands [5]. An early study from a single district in South Africa, at the time of Omicron emergence, suggested increased rates of COVID-19 hospitalization in children, but less severe illness than in previous waves, as indicated by reduced oxygen requirements and shorter hospital stays [14]. Data on COVID-19 epidemiology in children by fine age bands from African and low-middle-income settings spanning multiple waves are limited. We aimed to address this gap by providing data on national-level trends in SARS-CoV-2 in South Africa spanning four epidemic waves and including data from children in fine age bands. We describe the changing testing patterns and evolving epidemiology of diagnosed SARS-CoV-2 infections, hospitalizations, and deaths in children and adolescents aged ≤18 years in South Africa over four successive waves of infection and compare these to trends in adults. We also compare the severity of illness among hospitalized patients between the third (Delta) and fourth (Omicron BA.1 and BA.2) waves in different age groups.

## METHODS

### Data Sources

We used data from two national surveillance programs, the laboratory-based COVID-19 Notifiable Medical Conditions Surveillance System (NMCSS) and the DATCOV COVID-19 Hospital Surveillance system (DATCOV). Both programs generate national datasets for South Africa and have been previously described [15]. NMCSS includes all negative and positive test results on antigen and reverse-transcription real-time polymerase chain reaction (rRT-PCR) tests performed for SARS-CoV-2 [15, 16]. DATCOV includes all laboratory-confirmed hospitalized cases of COVID-19 from public and private hospitals throughout South Africa including nosocomial SARS-CoV-2 infection, or those that tested positive incidentally when admitted for other reasons [16]. Data from repeat infection episodes for both cases and hospitals were not available at the time of analysis. At the start of the pandemic in South Africa, SARS-CoV-2 testing was by rRT-PCR, with some limitations in testing availability. rRT-PCR data are transferred electronically to the National Institute for Communicable Diseases with data linkage. Testing for SARS-CoV-2 using rapid antigen-based tests started at the end of October 2020 [17]. Data on national SARS-CoV-2 laboratory testing, laboratory-confirmed cases, associated hospitalizations, and associated deaths were extracted from these surveillance systems on 15 March 2022 and the results reported were censored to include cases diagnosed until the end of the fourth (Omicron BA.1 and BA.2) wave on 5 February 2022, allowing at least 5 weeks for severe outcomes to accrue in diagnosed cases. Data on COVID-19 vaccination status were not available at the time of analysis. Vaccination was rolled out nationally to individuals aged >65 years from 17 March 2021 and progressively to younger individuals, becoming available to adolescents aged 12–17 years on 20 October 2021. As of June 2022, vaccination was not approved for children under 12 years of age in South Africa.

### Definitions of Study Population and Outcomes

Children and adolescents were defined as individuals aged ≤18 years, while adults were defined as individuals aged >18 years. A COVID-19 laboratory-confirmed case was defined as any person who tested positive for SARS-CoV-2 by rRT-PCR or an antigen test on a respiratory sample. COVID-19-associated hospital admissions were defined as any person who tested positive for SARS-CoV-2 and was admitted to the hospital regardless of the reason for admission. A COVID-19-associated in-hospital death was considered as any person who died in a hospital with a positive SARS-CoV-2 test from 1 March 2020 to 5 February 2022. Severe disease was defined as a person with a COVID-19-associated hospital admission who was admitted into high care or intensive care wards, received oxygen or invasive ventilation, developed acute respiratory distress syndrome, or died. The wave periods were defined as periods during which the weekly incidence risk was 30 cases or more per 100,000 population [18]. In South Africa, the first (Wuhan-Hu variant) wave occurred from weeks 24 to 34 of 2020, the second (Beta variant) wave from week 47 of 2020 to week 5 of 2021, the third (Delta variant) wave from weeks 19 to 37 of 2021, and the fourth (Omicron BA.1 and BA.2 subvariants) wave from week 48 of 2021 to week 5 of 2022.

### Statistical Analysis

Testing, case, admission, and death data from NMCSS and DATCOV were exported into Stata14.2 for analysis. The testing, case, admission, and death numbers and proportions were used to describe the study population by age groups (<1, 1–4, 5–12, 13–18, and >18 years). Age groups were chosen to distinguish between infants, toddlers, and primary and secondary school-attending ages in South Africa [19]. Weekly incidence rates were calculated per 100,000 population by age group and week of testing/positive test result/admission using the Statistics South Africa (Stats SA) 2020 and 2021 mid-year population estimates [20]. The in-hospital case-fatality ratio was calculated as a percentage of COVID-19-associated admissions with available outcomes, whose outcome was death during their hospital stay. Multivariable logistic regression was used to determine the odds of severe disease in the fourth wave compared to the third wave among individuals of all ages and separately in each age stratum after adjusting for sex, comorbidities, province, and health sector of admitting hospital. The adjusted odds ratios and 95% confidence intervals were reported. Variables for adjustment were selected based on data availability and included variables found to be associated with the outcome of interest in previous analyses [2, 16].

### Ethics

The Human Research Ethics Committee of the University of the Witwatersrand granted ethical approval for the collection of COVID-19 case and test data as part of communicable disease surveillance (M210752), and the DATCOV surveillance program (M2010108).

## RESULTS

### Overall Testing, Percent Testing Positive (PTP), Case and Admission Rates

From 1 March 2020 through 5 February 2022 in South Africa, children and adolescents aged ≤18 years accounted for 34.9% (21,008,060/60,142,978) of the population but only 13.6% (3,006,244/22,105,970) of all tests, 11.8% (424,394/3,593,644) cases, 5.8% (26,176/451,753) of admissions, and 0.7% (736/100,493) of in-hospital deaths. The overall in-hospital case-fatality ratio in individuals aged ≤18 years and with complete follow-up history was 2.9% (736/25,426), eight times lower than that of adults at 23.9% (99,757/417,460) (Table 1). The cumulative testing rate in individuals aged ≤18 years was 14,310 per 100,000 population over the study period, three times lower than the rate for adults, with the highest testing rate in infants, followed by adolescents aged 13–18 years. The cumulative case rate in individuals aged ≤18 years was 2,020 per 100,000 population, four times lower than that of adults. The cumulative admission rate in children aged ≤18 years was 125 per 100,000 population, eight times lower than that of adults. Among individuals aged ≤18 years, infants had the highest cumulative admission rate, followed by adolescents aged 13–18 years.

**Table 1.** Total Numbers and Cumulative Incidence of Individuals Tested for SARS-CoV-2, Laboratory-Confirmed Cases, Admissions, and In-hospital Deaths in Children and Adolescents Aged ≤18 Years and Individuals Aged >18 Years, 2020–2022, South Africa

Age Group (Years)	2021 Mid-Year Population Estimates, *n* (%)	Tests, *n* (%)	Cumulative Testing Rate/100,000 Population	Cumulative Percent Testing Positive (%)	Laboratory-Confirmed Cases, *n* (%)	Cumulative Laboratory-Confirmed Case Rate/100,000 Population	Admissions, *n* (%)	Cumulative Admission Rate/100,000 population	Deaths, *n* (%)	In-hospital Case-fatality Ratio (%)*
<1	1,175,632 (2.0)	477,677 (2.2)	40,632	11.3	11,451 (0.3)	974	5,938 (1.3)	505	267 (0.3)	4.7
1–4	4,533,324 (7.5)	457,907 (2.1)	10,101	8.6	43,233 (1.2)	954	5,570 (1.2)	123	89 (0.1)	1.6
5–12	9,127,182 (15.2)	910,808 (4.1)	9,979	16.7	146,963 (4.1)	1,610	5,735 (1.3)	63	112 (0.1)	2.0
13–18	6,171,922 (10.3)	1,159,852 (5.3)	18,792	20.2	222,747 (6.2)	3,609	8,933 (2.0)	145	268 (0.3)	3.1
>18	39,134,918 (65.1)	19,099,726 (86.4)	48,805	18.0	3,169,250 (88.2)	8,098	425,577 (94.2)	1,088	99,757 (99.3)	23.9
All ages	60,142,978 (100)	22,105,970 (100)	36,756	17.1	3,593,644 (100)	5,975	451,753 (100)	751	100,493 (100)	22.7

*CFR calculated for those with complete follow-up data (442,886). 5840 (0.03%) tests, 34,154 (0.9%) positive cases, 1600 (0.4%) admissions, and 108 (0.1%) deaths had unknown ages.

### Changes in Testing, PTP, Case, and Admission Rates by Wave Among All Age Groups

**Figure 1.**
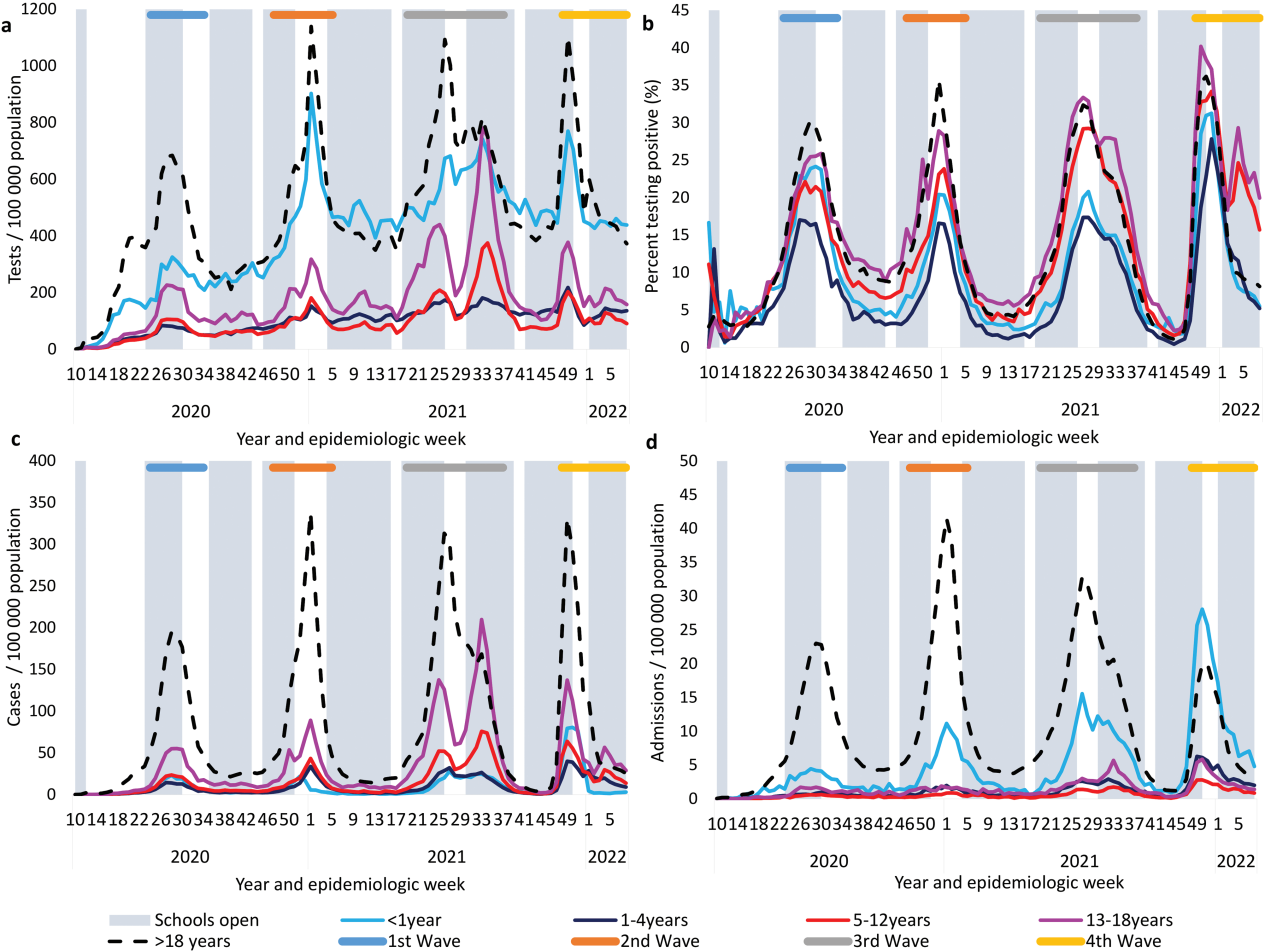
SARS-CoV-2 (a) testing rate, (b) percent testing positive, (c) laboratory-confirmed case rate, and (d) hospital admission rate per 100,000 population by epidemiologic week (e-h), South Africa, 1 March 2020 to 5 February 2022. Error bars specify 95% CI. CI, confidence interval.

**Figure 2.**
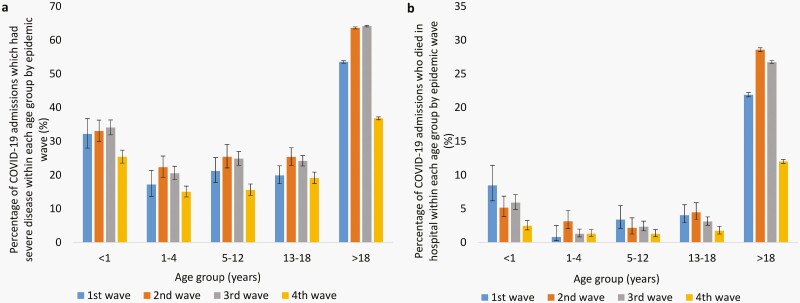
Percentage of COVID-19 admissions (a) who had severe disease and (b) who died in hospital within each age group by epidemic wave, South Africa, DATCOV, 1 March 2020 to 5 February 2022. Error bars specify 95% CI. CI, confidence interval.

The testing, case, and admission rate increased successively in the second (Beta) and third (Delta) waves among all age groups including adults, except for the case rate in infants which was lower in the second wave than in the first (Wuhan-Hu) (Supplementary Figure 1, panels a and b). In the fourth (Omicron BA.1 and BA.2) wave compared to the third (Delta) wave, the testing rate decreased among all age groups, while the case rate was higher among individuals aged <1 year and decreased among individuals aged ≥1 year. In the fourth wave, the admission rate increased among children aged <5 years, remained stable in children aged 5–12 years, and decreased in individuals aged ≥13 years (Supplementary Figure 1, panels a and b and Supplementary Figure 2). Among the children and adolescents aged ≤18 years, the admission rate was consistently highest in infants during all waves. The PTP dropped in all age groups in the second wave but increased in individuals aged 1–18 years in the third wave and among all age groups in the fourth wave. In the third and fourth waves, weekly PTP and case rates in children of school-going age (5–18 years) increased noticeably with rates in 13- to 18-year-olds exceeding rates among those >18 years in the second half of the third and fourth waves (Figure 1a–d). In the first three waves, weekly admission rates were consistently highest in adults, followed by infants; but in the fourth wave, weekly admission rates in infants increased, exceeding rates in adults (which were lower in the fourth wave compared to previous waves). Weekly hospitalization rates were also increased in children aged 1–4 years in the fourth wave compared to previous waves (Figure 1a–d). Although rates of hospitalization were increased in the fourth wave among children aged <5 years, the proportion of admitted cases with severe COVID-19 was lower in all age groups (Figure 2a). The in-hospital case-fatality ratio was also lower in the fourth wave cumulatively and compared to previous waves, in all age groups except for the 1- to 4-year-olds, where it was similar to the third wave, but numbers were low (Figure 2b).

### Proportion of Admissions with Severe Illness in the Fourth Wave vs the Third Wave

Lower odds of severe outcomes or illness in hospitalized individuals were observed in the fourth wave compared to the third wave overall and within all age groups with the magnitude of the reduction in severity greater among individuals aged >18 years than those ≤18 years (Table 2). In each age group and overall, the odds of severe disease were approximately two times higher in those with ≥1 comorbidity than in those without (Supplementary Tables 1–6).

**Table 2.** Multivariable Analysis of the Proportion of SARS-CoV-2 Admissions which were Severe in the Fourth Wave Compared to the Third Wave in Different Age Groups, 2020–2022, South Africa

Age Group	Severe Disease Wave 3	Severe Disease Wave 4	Unadjusted OR	Adjusted OR^a^
<1 year	611/1,792 (34.1)	507/1,995 (25.4)	0.7 (0.6–0.8)***	0.6 (0.6–0.7)***
1–4 years	321/1,562 (20.6)	289/1,923 (15.0)	0.7 (0.6–0.8)***	0.7 (0.6–0.8)***
5–12 years	437/1,758 (24.9)	282/1,811 (15.6)	0.6 (0.5–0.7)***	0.6 (0.5–0.7)***
13–18 years	723/2,989 (24.2)	399/2,085 (19.1)	0.7 (0.6–0.9)***	0.7 (0.6–0.9)***
>18 years	89,135/138,937 (64.2)	17,993/48,881 (36.8)	0.3 (0.3–0.3)***	0.3 (0.3–0.3)***
All ages	91,227/147,038 (62.0)	19,470/56,695 (34.3)	0.3 (0.3–0.3)***	0.3 (0.3–0.3)***

***p < 0.001; OR—odds ratio.

^a^Modeling odds of severe disease adjusted for sex, comorbidities, province, and hospital sector. Separate multivariable models were constructed for each age group. Full multivariable models for each age group are presented in the supplement.

## DISCUSSION

In this study of COVID-19 in children and adolescents in South Africa, we found that following four waves, individuals aged ≤18 years accounted for 13.6% of the total SARS-CoV-2 tests, 11.8% of laboratory-confirmed cases, 5.8% of COVID-19 associated hospital admissions, and 0.7% of COVID-19 associated deaths, despite comprising almost 35% of the population. While the rate of tests, PTP, cases, and admissions was lower among children during the first SARS-CoV-2 wave, successive waves saw a shift to proportionately more testing, diagnosed cases, and admissions in children. In the fourth (Omicron BA.1 and BA.2) wave, the rates of hospitalization increased among children aged <5 years and severity was lower among hospitalized individuals.

Increased diagnosed cases among children, possibly driven in part due to the increase in testing, especially in the Delta and Omicron waves have been observed in various countries, including the United States and the United Kingdom [9, 10]. Other reasons could be the circulation of the highly transmissible Delta and Omicron variants at a time when COVID-19 restrictions were being eased, with relatively decreased diagnosed cases among adults following widespread vaccination or previous infection over successive waves. In our study, the testing rates for all age groups increased with each wave, and, as time progressed, more tests became available and the testing criteria also became less strict. Notably, the testing rates for the <1-year age group remained consistently higher than the other age groups ≤18 years and comparable to that of adults and possibly as a result of infants being more likely to be admitted to the hospital even if COVID-19 was not the reason for admission. Increased exposure to symptomatic adults or caregivers may have also been another reason.

We noted an increase in case rates among children of school-going age in the third and fourth waves, particularly when schools were open, this could be attributed to increased transmissibility of the Delta and Omicron variants, changes in school policies allowing for more in-person/contact teaching, increased testing of children in part due to these policy changes or increased engagement in social activities, unrelated to schooling [21–23]. According to the South African department of health, at the start of the fourth wave (week of 28 November 2021), 42% of the population aged ≥18 years had been vaccinated with at least one dose whereas 6% of those aged 12–17 years had received at least one dose of a SARS-CoV-2 vaccine [24]. Serologic studies after the second wave showed seroprevalence of 18%–53% among individuals ≤18 years versus 25%–59% in individuals >18 years after the second wave and 43%–83% versus 49%–79% after the third wave, suggesting that an immunity gap in children was likely not a major contributor to the shift in diagnosed case numbers. Importantly, based on serologic studies, <10% of all SARS-CoV-2 cases were diagnosed in South Africa as of November 2021, meaning that trends in case numbers may be substantially biased by changes in testing practice and most cases in children are undiagnosed [25].

The rate of COVID-19 hospital admissions increased through successive waves for children aged <5 years. In contrast, in the fourth wave, they remained stable in those aged 5–12 years and decreased in older individuals. Uniquely, in the fourth wave, the admission rate for infants increased to such an extent that it surpassed the rate for adults. Similar findings of increased admissions among children in the Omicron wave were previously reported from a provincial district in South Africa and the United Kingdom [10, 14, 26]. Other non-COVID-19 respiratory viruses such as influenza and respiratory syncytial virus (RSV) were in circulation prior to the onset of the fourth wave, therefore a proportion of the observed hospital admissions could have potentially resulted from infection with other viruses, with incidental positive tests for SARS-CoV-2 [27]. Importantly, while hospital admission rates were higher among young children, we also demonstrated that in-hospital severity was lower. This is consistent with a detailed report of clinical and in-hospital outcomes of children hospitalized early in the fourth wave in an area of South Africa in which COVID-19 was the primary diagnosis in only 44% of cases, 88% required only general care, 20% required oxygen, the median duration of hospitalization was 2 days, and the in-hospital mortality was 0% in those with COVID-19 attributable hospitalization [14]. Studies from the United States and other countries also described milder illness among children during the Omicron wave [4, 14, 28]. In our study, we found an overall in-hospital CFR of 3% in children from the period 1 March 2020 to 5 February 2022 which was lower than a study done in the Democratic Republic of Congo reporting a CFR of 11.8% and a multicountry sub-Saharan Africa cohort study reporting 8.3% [29, 30]. The overall COVID-19 in-hospital CFR of 3.2% in children < 5 years, was higher than historical estimates of CFR from South African sentinel surveillance among children hospitalized with influenza (1.0%) or RSV (0.8%), suggesting that COVID-19 in young children can be more severe compared to other respiratory viruses [31]. In all age strata studied, children with underlying comorbidities had approximately two times greater odds of severe illness. This is similar to previous studies from other settings [6, 32].

This study was conducted on a nationwide scale, with high numbers of children tested, infected, admitted, and followed for outcomes, to describe epidemiological patterns and trends of COVID-19 in South African children and adolescents. This study is notable for reporting findings spanning four epidemic and interwave periods rather than one, and it is based on an additional 19 million tests compared to a previous 2020 study of COVID-19 in South African children, as well as an additional 370,000 cases, 115,000 admissions, and 20,000 fatalities compared to a previous clinical severity study in all reported COVID-19 cases and hospitalizations [2, 5]. However, there were some limitations. The differing testing strategies and recommendations over time and by the health sector, varying access to testing between private and public healthcare sectors, availability of tests, and/or reporting of tests may inadvertently alter the number of tests, PTP, and case rate. Because the case data are dependent on individuals presenting themselves to testing facilities, there was likely substantial under-ascertainment of cases, particularly among asymptomatic or non-health-seeking individuals. Differences in healthcare-seeking behavior by age and gender also have an impact on case data, as asymptomatic people have no reason to seek medical attention [33]. The hospital admission data are incomplete in terms of the reasons for admission, prior infections and underlying conditions, resulting in these not being adjusted for in the analyses and thereby potentially biasing the results. Furthermore, detailed data on treatment and COVID-19 vaccination information were not available, limiting inferences on the severity of the disease. Also, the number of deaths being reported may be an underestimation as these only account for in-hospital deaths and not deaths occurring outside of health facilities. The lack of key information from the data sources limits our ability to attribute the admissions or deaths to SARS-CoV-2 infection.

In conclusion, in South Africa, the testing and admission rates among children have been increasing with the Beta and Delta waves. In the Omicron wave, hospitalization rates increased among children aged <5 years but case and hospitalization rates decreased in older children and adults. Infants had the highest rates for testing, admission, and in-hospital case-fatality in all waves including the fourth (Omicron BA.1 and BA.2) wave. Furthermore, SARS-CoV-2 was associated with higher in-hospital case-fatality ratios than influenza or RSV in hospitalized South African children <5 years [31]. Importantly, even though children <5 years had the highest admission rates in the fourth wave, they still had less severe outcomes. Children and adolescents who had at least one comorbidity had higher odds of severe outcomes than those without, therefore, vaccination should be considered to be expanded to children aged 5–11 years with underlying illness.

## Supplementary Material

piad002_suppl_Supplementary_Figure_S1

piad002_suppl_Supplementary_Figure_S2

piad002_suppl_Supplementary_Tables

## Data Availability

Data used in this manuscript are available upon reasonable request. Proposals should be directed to cherylc@nicd.ac.za.

## Notes

***Financial support.*** This work was supported by grants from the National Institute for Communicable Diseases and the National Department of Health, Republic of South Africa.

***Potential conflicts of interest.*** CC has received grant support from Sanofi Pasteur, US CDC, Welcome Trust, Program for Applied Technologies in Health, Bill & Melinda Gates Foundation, and South African Medical Research Council (SA-MRC). AvG and NW have received grant support from Sanofi Pasteur and the Gates Foundation. MG has received grants from Bill & Melinda Gates Foundation and South African Medical Research Council. The remaining authors declare no conflict of interest.
