# Supplementary figures and images for: Changing Epidemiology of COVID-19 in Children and Adolescents Over Four Successive Epidemic Waves in South Africa, 2020–2022
Source: J Pediatric Infect Dis Soc. 2023 Jan 17;12(3):128–34. doi: 10.1093/jpids/piad002 (PMC10112681; doi:10.1093/jpids/piad002)

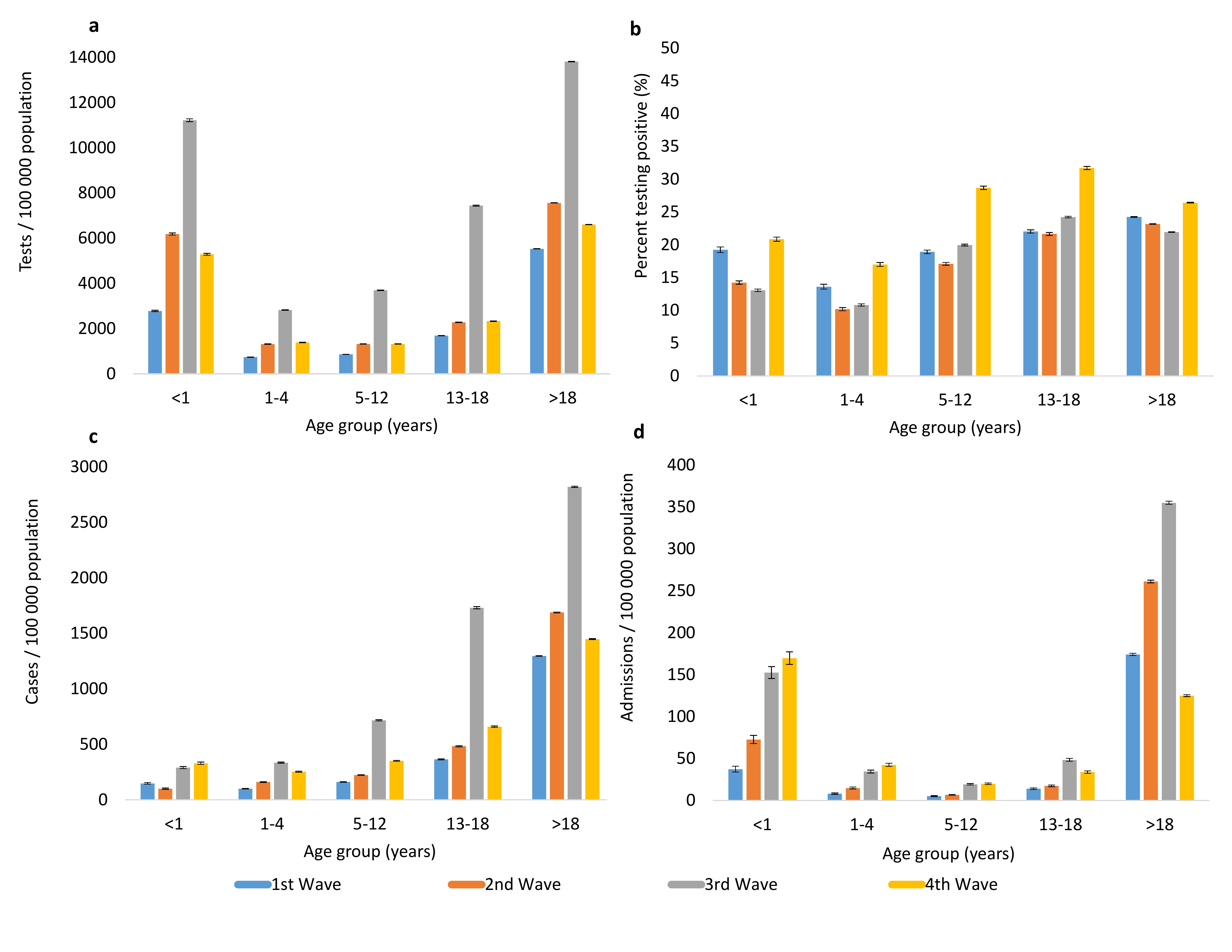

Supplement: piad002_suppl_Supplementary_Figure_S1 [file piad002_suppl_supplementary_figure_s1.jpeg]

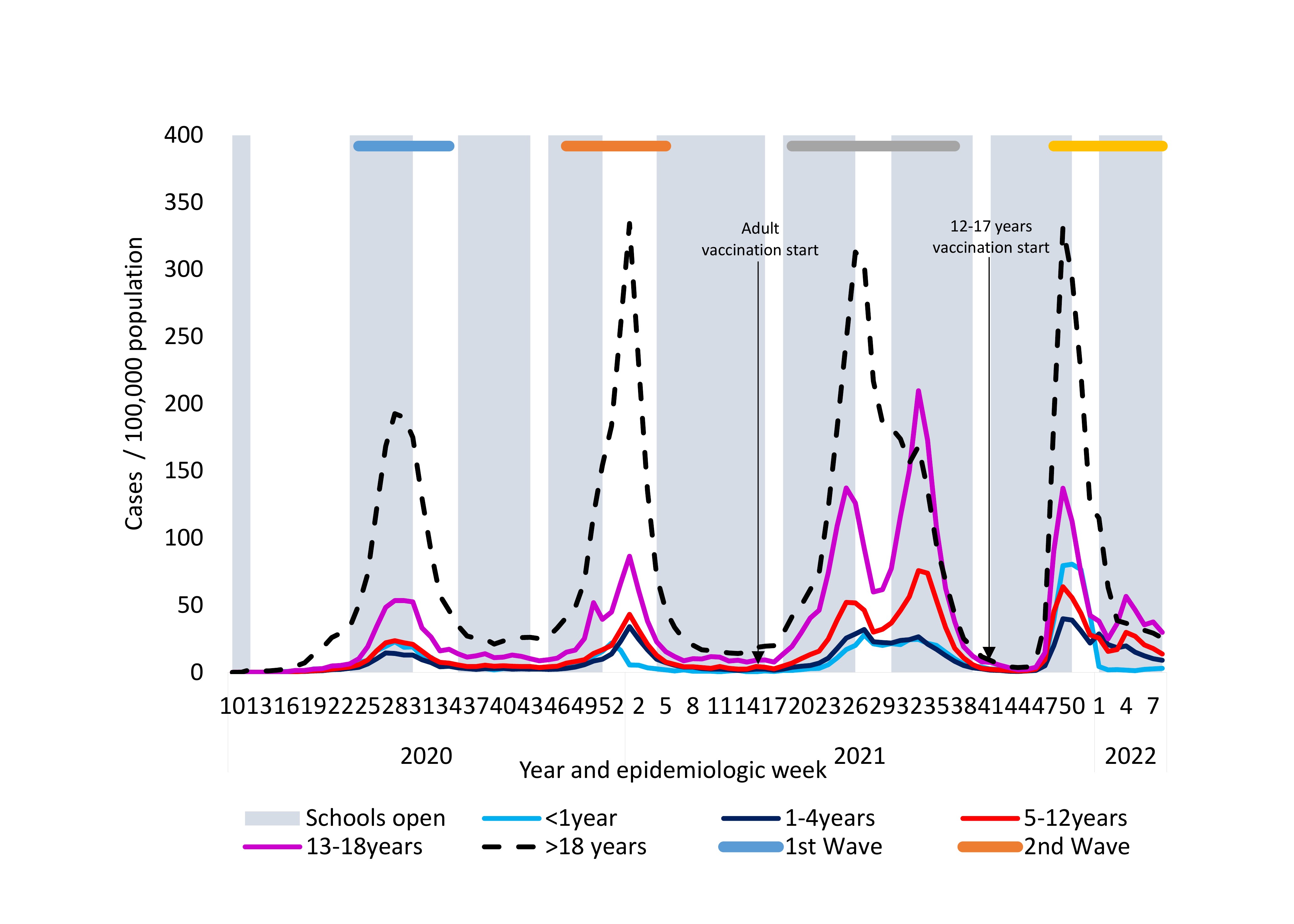

Supplement: piad002_suppl_Supplementary_Figure_S2 [file piad002_suppl_supplementary_figure_s2.jpeg]
