# Supplementary material for: Changing Epidemiology of COVID-19 in Children and Adolescents Over Four Successive Epidemic Waves in South Africa, 2020–2022
Source: J Pediatric Infect Dis Soc. 2023 Jan 17;12(3):128–34. doi: 10.1093/jpids/piad002 (PMC10112681; doi:10.1093/jpids/piad002)
Supplement: piad002_suppl_Supplementary_Tables [file piad002_suppl_supplementary_tables.docx]

**Supplementary Table 1.** Complete multivariable models of SARS-CoV-2 admissions which were severe in the fourth wave compared to the third wave in those <1 year, 2020-2022, South Africa.

| **<1 year** |  | Adjusted odds ratio | 95% confidence interval | p-value |
| --- | --- | --- | --- | --- |
| Epidemic wave | Wave 3 | Reference |  |  |
|  | Wave 4 | 0.6 | (0.6-0.7) | <0.001 |
| Gender | Female | Reference |  |  |
|  | Male | 1.1 | (0.9-1.2) | 0.440 |
| Province | Eastern Cape | 1.2 | (0.8-1.7) | 0.399 |
|  | Free State | 1.2 | (0.9-1.8) | 0.249 |
|  | Gauteng | 1.2 | (0.9-1.4) | 0.166 |
|  | KwaZulu-Natal | 0.7 | (0.5-0.9) | 0.003 |
|  | Northern Cape | 1.5 | (0.8-2.8) | 0.264 |
|  | Western Cape | 0.4 | (0.3-0.5) | <0.001 |
|  | Other provinces | Reference |  |  |
| Hospital sector | Private | Reference |  |  |
|  | Public | 1.6 | (1.4-1.8) | <0.001 |
| One or more comorbidities | No | Reference |  |  |
|  | Yes | 1.6 | (1.2-2.2) | 0.001 |

**Supplementary Table 2.** Complete multivariable models of SARS-CoV-2 admissions which were severe in the fourth wave compared to the third wave in those 1-4 years, 2020-2022, South Africa.

| **1-4 years** |  | Adjusted odds ratio | 95% confidence interval | p-value |
| --- | --- | --- | --- | --- |
| Epidemic wave | Wave 3 | Reference |  |  |
|  | Wave 4 | 0.7 | (0.6-0.8) | <0.001 |
| Gender | Female | Reference |  |  |
|  | Male | 1.1 | (0.9-1.3) | 0.529 |
| Province | Eastern Cape | 0.8 | (0.5-1.3) | 0.419 |
|  | Free State | 1.4 | (0.9-2.2) | 0.111 |
|  | Gauteng | 1.3 | (1.0-1.6) | 0.079 |
|  | KwaZulu-Natal | 0.7 | (0.5-1.0) | 0.033 |
|  | Northern Cape | 0.6 | (0.3-1.3) | 0.200 |
|  | Western Cape | 0.3 | (0.2-0.5) | <0.001 |
|  | Other provinces | Reference |  |  |
| Hospital sector | Private | Reference |  |  |
|  | Public | 2.3 | (1.9-2.8) | <0.001 |
| One or more comorbidities | No | Reference |  |  |
|  | Yes | 1.6 | (1.2-2.2) | 0.001 |

**Supplementary Table 3.** Complete multivariable models of SARS-CoV-2 admissions which were severe in the fourth wave compared to the third wave in those 5-12 years, 2020-2022, South Africa.

| **5-12 years** |  | Adjusted odds ratio | 95% confidence interval | p-value |
| --- | --- | --- | --- | --- |
| Epidemic wave | Wave 3 | Reference |  |  |
|  | Wave 4 | 0.6 | (0.5-0.7) | <0.001 |
| Gender | Female | Reference |  |  |
|  | Male | 1.0 | (0.8-1.1) | 0.594 |
| Province | Eastern Cape | 1.0 | (0.7-1.5) | 0.986 |
|  | Free State | 1.3 | (0.9-1.9) | 0.170 |
|  | Gauteng | 1.6 | (1.3-2.1) | <0.001 |
|  | KwaZulu-Natal | 0.9 | (0.7-1.2) | 0.452 |
|  | Northern Cape | 0.7 | (0.4-1.3) | 0.213 |
|  | Western Cape | 0.4 | (0.3-0.6) | <0.001 |
|  | Other provinces | Reference |  |  |
| Hospital sector | Private | Reference |  |  |
|  | Public | 2.1 | (1.8-2.6) | <0.001 |
| One or more comorbidities | No | Reference |  |  |
|  | Yes | 1.9 | (1.5-2.4) | <0.001 |

**Supplementary Table 4.** Complete multivariable models of SARS-CoV-2 admissions which were severe in the fourth wave compared to the third wave in those 13-18 years, 2020-2022, South Africa.

| **13-18 years** |  | Adjusted odds ratio | 95% confidence interval | p-value |
| --- | --- | --- | --- | --- |
| Epidemic wave | Wave 3 | Reference |  |  |
|  | Wave 4 | 0.7 | (0.6-0.9) | <0.001 |
| Gender | Female | Reference |  |  |
|  | Male | 1.1 | (1.0-1.3) | 0.141 |
| Province | Eastern Cape | 0.8 | (0.6-1.1) | 0.216 |
|  | Free State | 1.5 | (1.1-1.9) | 0.009 |
|  | Gauteng | 1.5 | (1.2-1.8) | <0.001 |
|  | KwaZulu-Natal | 0.9 | (0.7-1.1) | 0.442 |
|  | Northern Cape | 1.0 | (0.7-1.6) | 0.825 |
|  | Western Cape | 0.5 | (0.4-0.7) | <0.001 |
|  | Other provinces | Reference |  |  |
| Hospital sector | Private | Reference |  |  |
|  | Public | 1.2 | (1.1-1.4) | 0.008 |
| One or more comorbidities | No | Reference |  |  |
|  | Yes | 2.2 | (1.8-2.6) | <0.001 |

**Supplementary Table 5.** Complete multivariable models of SARS-CoV-2 admissions which were severe in the fourth wave compared to the third wave in those >18 years, 2020-2022, South Africa.

| **>18 years** |  | Adjusted odds ratio | 95% confidence interval | p-value |
| --- | --- | --- | --- | --- |
| Epidemic wave | Wave 3 | Reference |  |  |
|  | Wave 4 | 0.3 | (0.3-0.3) | <0.001 |
| Gender | Female | Reference |  |  |
|  | Male | 1.4 | (1.4-1.4) | <0.001 |
| Province | Eastern Cape | 1.4 | (1.3-1.4) | <0.001 |
|  | Free State | 1.4 | (1.3-1.4) | <0.001 |
|  | Gauteng | 1.0 | (1.0-1.0) | 0.301 |
|  | KwaZulu-Natal | 0.8 | (0.8-0.8) | <0.001 |
|  | Northern Cape | 1.4 | (1.3-1.5) | <0.001 |
|  | Western Cape | 0.4 | (0.4-0.4) | <0.001 |
|  | Other provinces | Reference |  |  |
| Hospital sector | Private | Reference |  |  |
|  | Public | 0.7 | (0.7-0.8) | <0.001 |
| One or more comorbidities | No | Reference |  |  |
|  | Yes | 2.1 | (2.0-2.1) | <0.001 |

**Supplementary Table 6.** Complete multivariable models of SARS-CoV-2 admissions which were severe in the fourth wave compared to the third wave in all ages, 2020-2022, South Africa.

| **All ages** |  | Adjusted odds ratio | 95% confidence interval | p-value |
| --- | --- | --- | --- | --- |
| Epidemic wave | Wave 3 | Reference |  |  |
|  | Wave 4 | 0.3 | (0.3-0.3) | <0.001 |
| Gender | Female | Reference |  |  |
|  | Male | 1.4 | (1.3-1.4) | <0.001 |
| Province | Eastern Cape | 1.3 | (1.3-1.4) | <0.001 |
|  | Free State | 1.4 | (1.3-1.5) | <0.001 |
|  | Gauteng | 1.1 | (1.0-1.1) | <0.001 |
|  | KwaZulu-Natal | 0.8 | (0.8-0.8) | <0.001 |
|  | Northern Cape | 1.4 | (1.3-1.5) | <0.001 |
|  | Western Cape | 0.4 | (0.4-0.4) | <0.001 |
|  | Other provinces | Reference |  |  |
| Hospital sector | Private | Reference |  |  |
|  | Public | 0.8 | (0.7-0.8) | <0.001 |
| One or more comorbidities | No | Reference |  |  |
|  | Yes | 2.3 | (2.2-2.3) | <0.001 |

**Supplementary Figure 1.** SARS-CoV-2 (a) testing rate, (b) percent testing positive (c) laboratory-confirmed case rate, and (d) hospital admission rate per 100,000 population by epidemic wave and age group, South Africa, 1 March 2020 – 5 February 2022. Error bars specify 95% CI. CI, confidence interval.

**Supplementary Figure 2.** SARS-CoV-2 laboratory-confirmed case rate per 100,000 population by epidemiologic week, South Africa, 1 March 2020 – 5 February 2022.
